# Supplementary material for: Clump sequencing exposes the spatial expression programs of intestinal secretory cells
Source: Nat Commun. 2021 May 24;12:3074. doi: 10.1038/s41467-021-23245-2 (PMC8144370; doi:10.1038/s41467-021-23245-2)
Supplement: Supplementary file 1 — Supplementary Information [file 41467_2021_23245_MOESM1_ESM.pdf]

Supplementary files for

Clump sequencing exposes the spatial expression programs of intestinal secretory cells

Rita Manco*, Inna Averbukh*, Ziv Porat, Keren Bahar Halpern, Ido Amit, Shalev Itzkovitz

Correspondence to: shalev.itzkovitz@weizmann.ac.il

This PDF file includes:

Supplementary figures 1 to 18

Other Supplementary Materials for this manuscript includes the following:

Supplementary Data 1 to 11 as Excel files

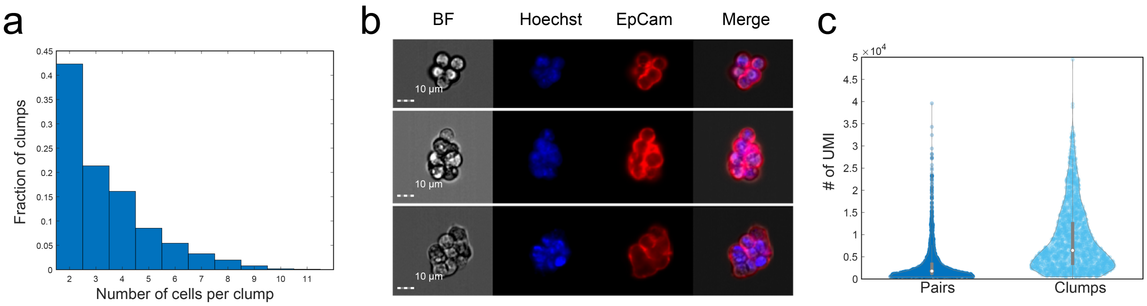

Supplementary Figure 1 (a) Quantification of the number of cells per clump, based on Imagestream images using Hoechst DNA nuclear staining. n = 100 clumps per mouse (n =3 mice). Source data are provided as a Source Data file. (b) Representative Imagestream images of clumps (n=3 mice). (c) Clumps with more than two cells have higher UMIs counts compare to clumps with 2 cells (pairs). Violin plot of total sum of UMI counts for pairs and larger clumps. n pairs = 2926, n clumps = 1862, examined over 5 independent experiments. White circles mark the medians, gray boxes mark the 25-75 percentiles.

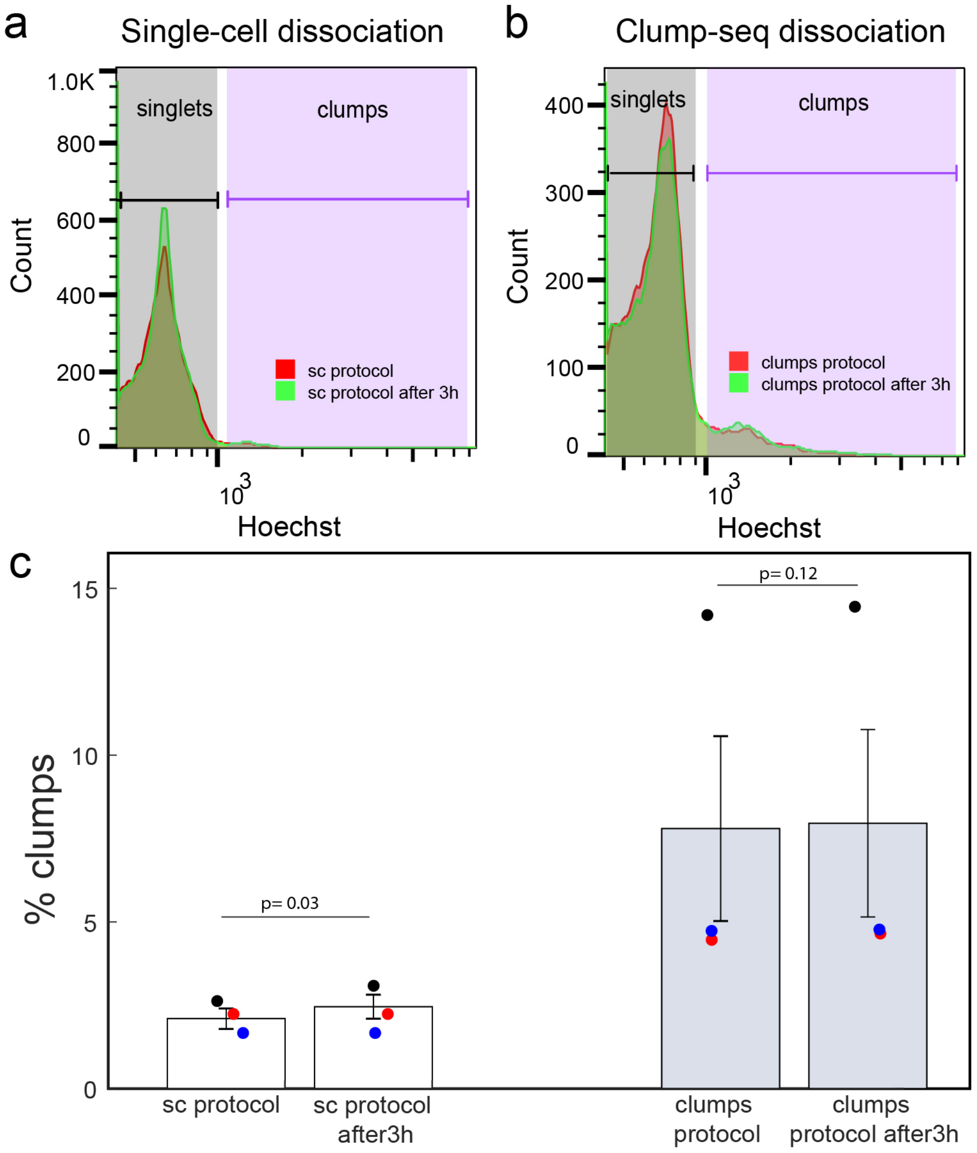

Supplementary Figure 2 Background analysis for dissociation protocol (a-b) Representative histograms of the Hoechst DNA content staining at time 0h (red curve) and 3 h (green curve) for the single-cell dissociation protocol (a) and clump dissociation protocol (b). (c) Quantification of clumps immediately after dissociation for single-cell and clumps protocol at time 0h and after 3h, n=3 mice, (sc protocol p value by t test (two-sided) = 0.03; clumps protocol p value by t test (two-sided) = 0.12). Data are presented as mean values +/- SD. Source data are provided as a Source Data file.

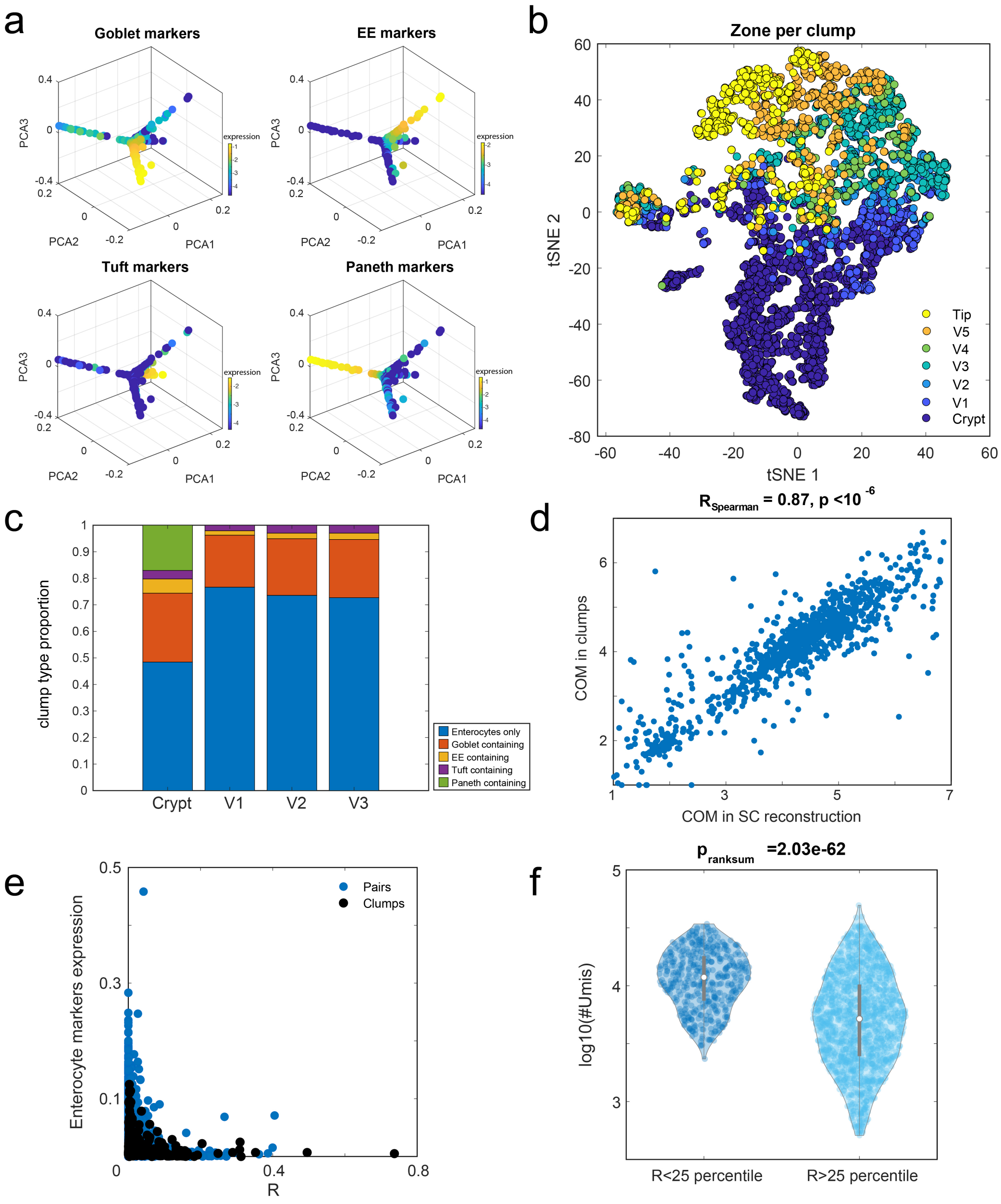

Supplementary Figure 3 (a) Scatter plot of pairs in 3D PC space of type markers sum (Methods, Supplementary Table 7), colored by classification markers sum. The pairs form four rays emanating from the origin of the axis. Along each of the rays, the marker sum of a single secretory cell type is highest and reaches a peak at the tip of the ray furthest from the origin. (b) tSNE plot of all clumps colored by their assigned zone. (c) Frequency of clump types in the crypt and 3 villus zones. The calculation was performed on the clumps zonation table Supplementary Data 2, coarse grained into 4 zones. (d) Validation for clumps zone assignment, by comparing center of mass (COM) of enterocyte specific genes in clumps based spatial reconstruction with single cell spatial reconstruction performed in Moor et al.[7] (Methods). p = 1e-307. (e) Summed expression of enterocyte type markers (Supplementary Data 7) as function of R, the distance from origin in PC space, for clumps and pairs (Methods). (f) Violin plots of total sum of UMIs per clump, for clumps within and outside the lower 25 percentile of R values. n pairs = 2926, n clumps = 1862, examined over 5 independent experiments. White circles mark the medians, gray boxes mark the 25-75 percentiles. P values were calculated with the Kruskal-Wallis test.

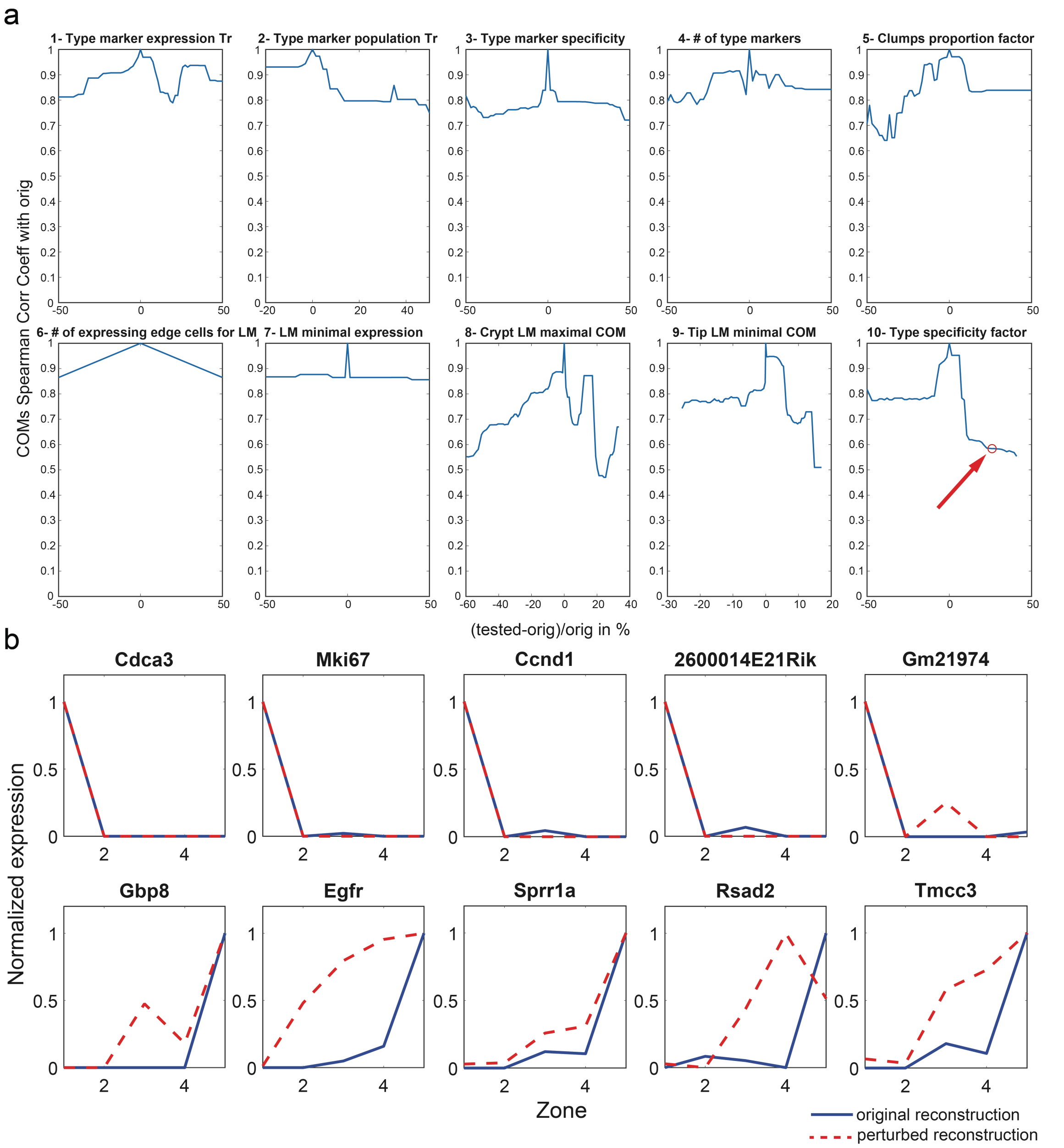

Supplementary Figure 4 Sensitivity analysis for goblet cell zonation reconstruction. (a) Correlations between zonation reconstructions as function of parameter perturbations. X axis indicates the distance between the original and perturbed parameter calculated as the difference between the two divided by the original value in percent. The Y axis indicates the Spearman correlation coefficient between the centers of masses (COMs) of the original reconstruction and perturbed reconstruction (See methods for details). Parameters are detailed in Supplementary Data 11. Red circle marked with red arrow indicates an example of a perturbed reconstruction for which correlation with the original reconstruction was lower. (b) Examples for individual zonation profiles in the original (blue lines) compared to the indicated perturbed reconstruction (dashed red lines). For this purpose, 5 tip and 5 crypt zonated, highly expressed genes not used as landmarks were selected (See methods).

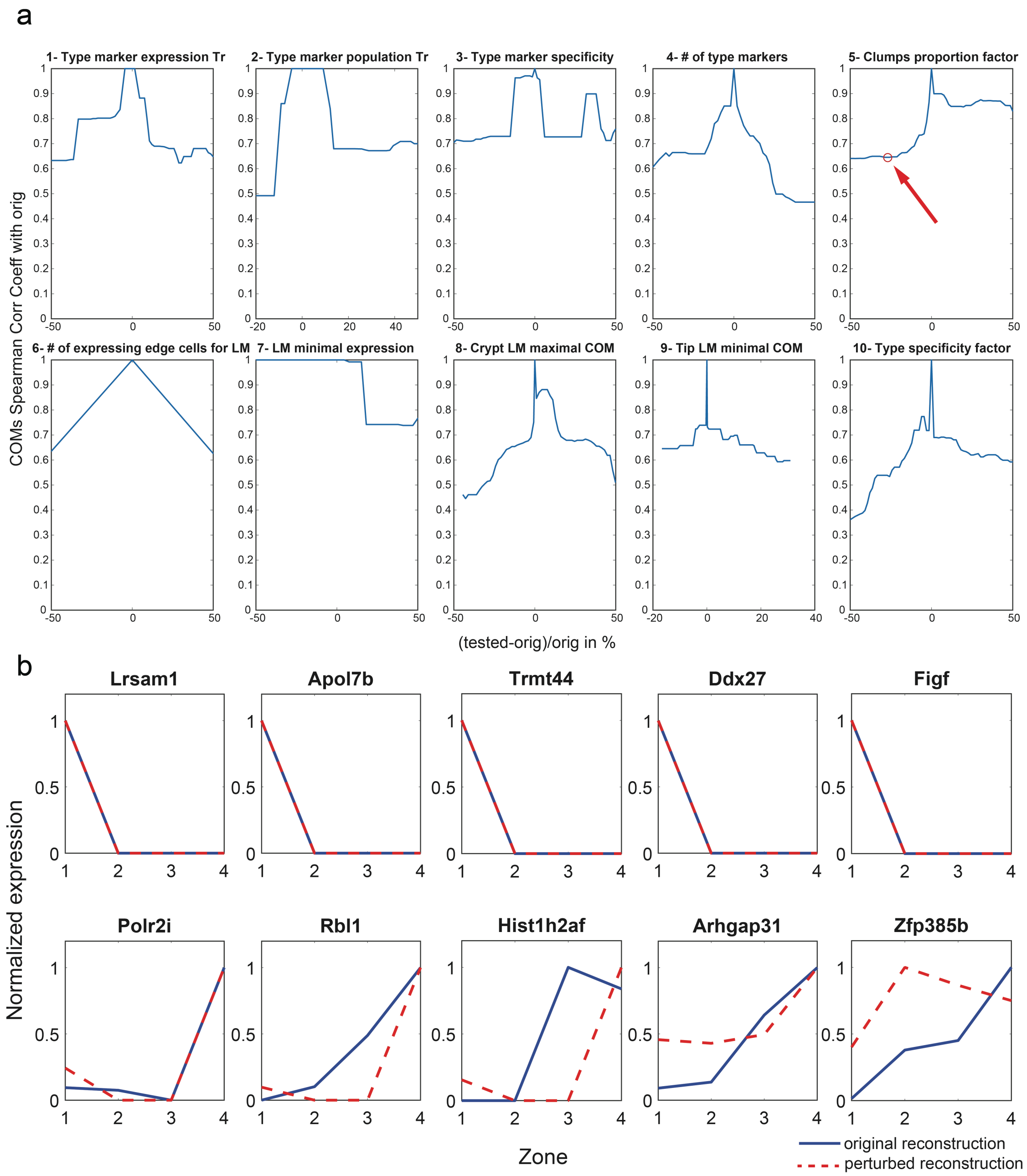

Supplementary Figure 5 Sensitivity analysis for tuft cell zonation reconstruction. (a) Correlations between zonation reconstructions as function of parameter perturbations. X axis indicates the distance between the original and perturbed parameter calculated as the difference between the two divided by the original value in percent. The Y axis indicates the Spearman correlation coefficient between the centers of masses (COMs) of the original reconstruction and perturbed reconstruction (See methods for details). Parameters are detailed in Supplementary Data 11. Red circle marked with red arrow indicates an example of a perturbed reconstruction for which correlation with the original reconstruction was lower. (b) Examples for individual zonation profiles in the original (blue lines) compared to the indicated perturbed reconstruction (dashed red lines). For this purpose, 5 tip and 5 crypt zonated, highly expressed genes not used as landmarks were selected (See methods).

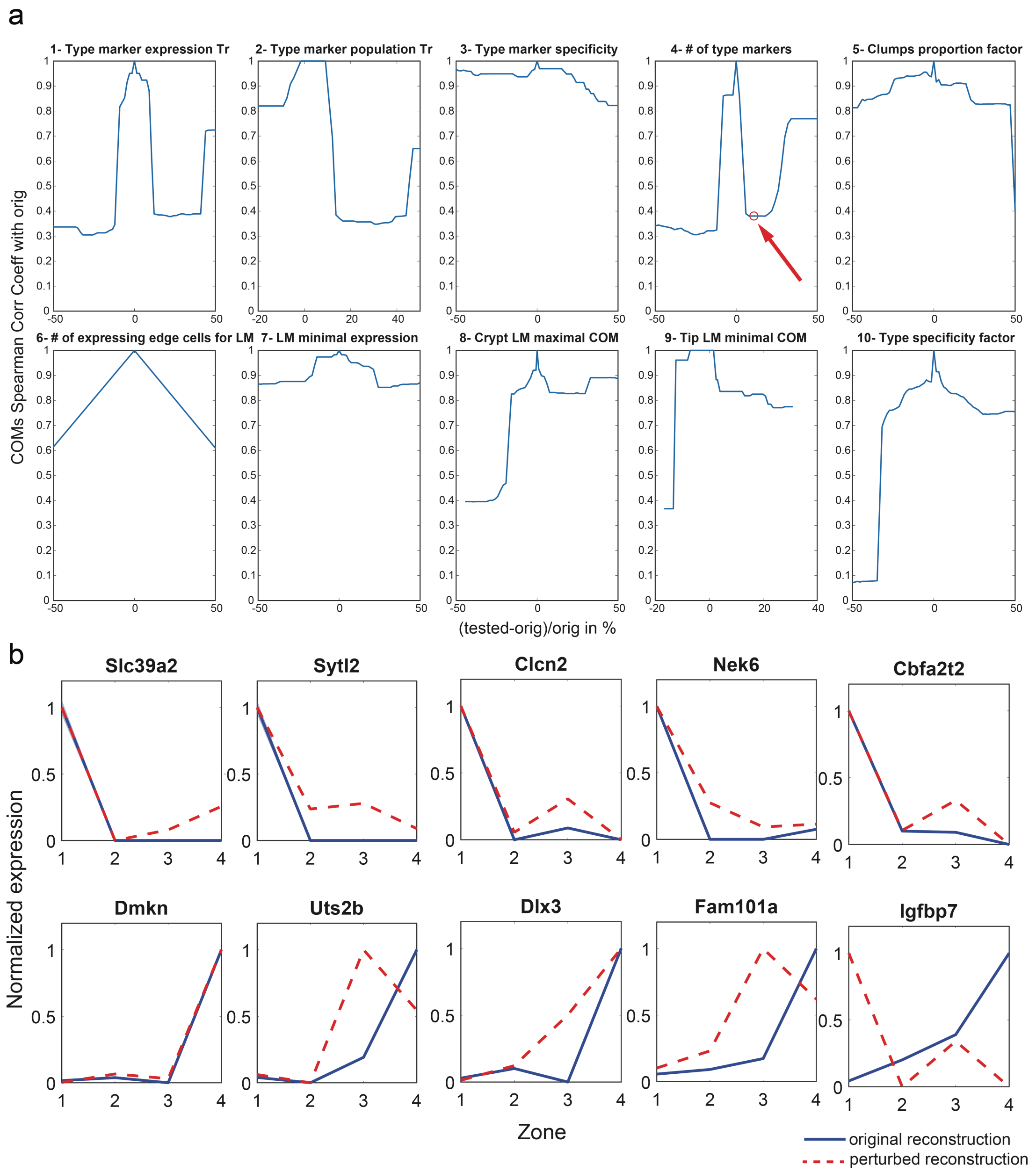

Supplementary Figure 6 Sensitivity analysis for enteroendocrine cell zonation reconstruction. (a) Correlations between zonation reconstructions as function of parameter perturbations. X axis indicates the distance between the original and perturbed parameter calculated as the difference between the two divided by the original value in percent. The Y axis indicates the Spearman correlation coefficient between the centers of masses (COMs) of the original reconstruction and perturbed reconstruction (See methods for details). Parameters are detailed in Supplementary Data 11. Red circle marked with red arrow indicates an example of a perturbed reconstruction for which correlation with the original reconstruction was lower. (b) Examples for individual zonation profiles in the original (blue lines) compared to the indicated perturbed reconstruction (dashed red lines). For this purpose, 5 tip and 5 crypt zonated, highly expressed genes not used as landmarks were selected (See methods).

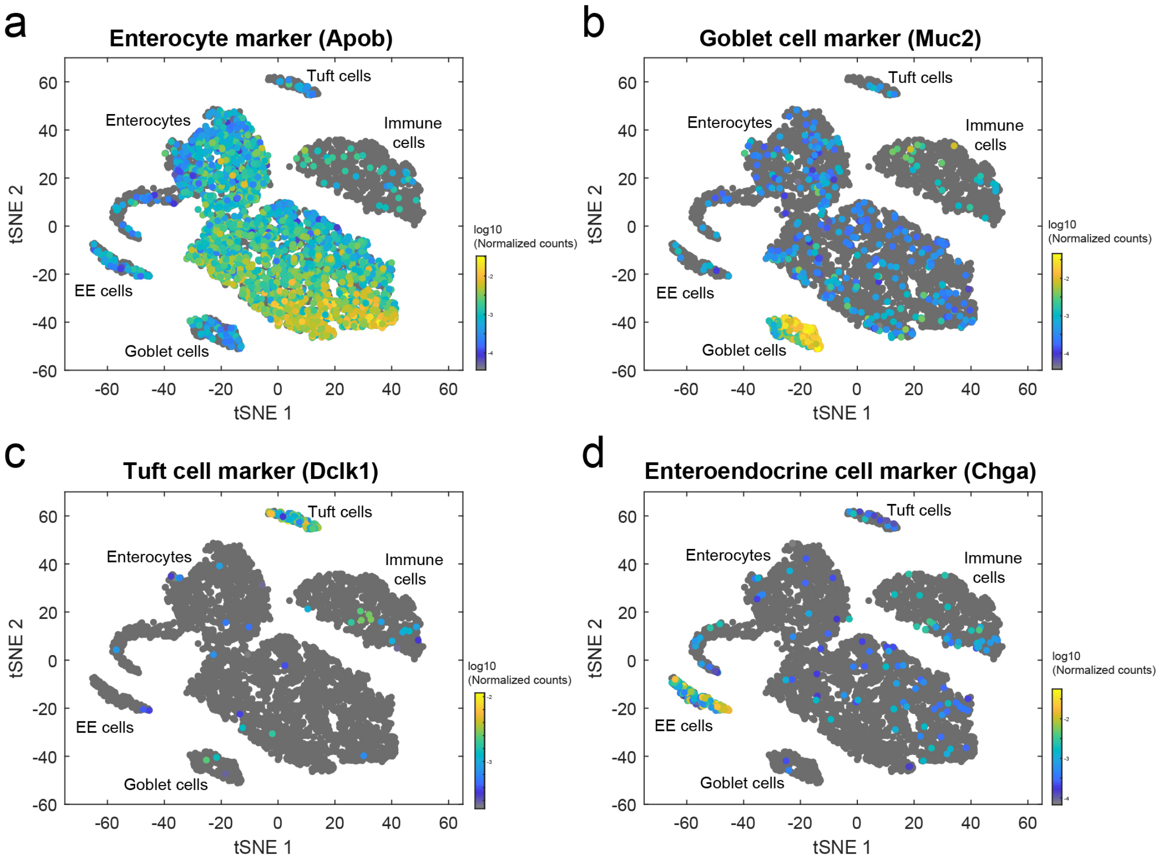

Supplementary Figure 7 tSNE plots showing the identified clusters from the single cell sequencing: (a) Enterocytes, colored by Apob, (b) Goblet cells, Muc2^{+}, (c) Tuft cells, Dclk1^{+} and (d) Enteroendocrine cells, Chga^{+}.

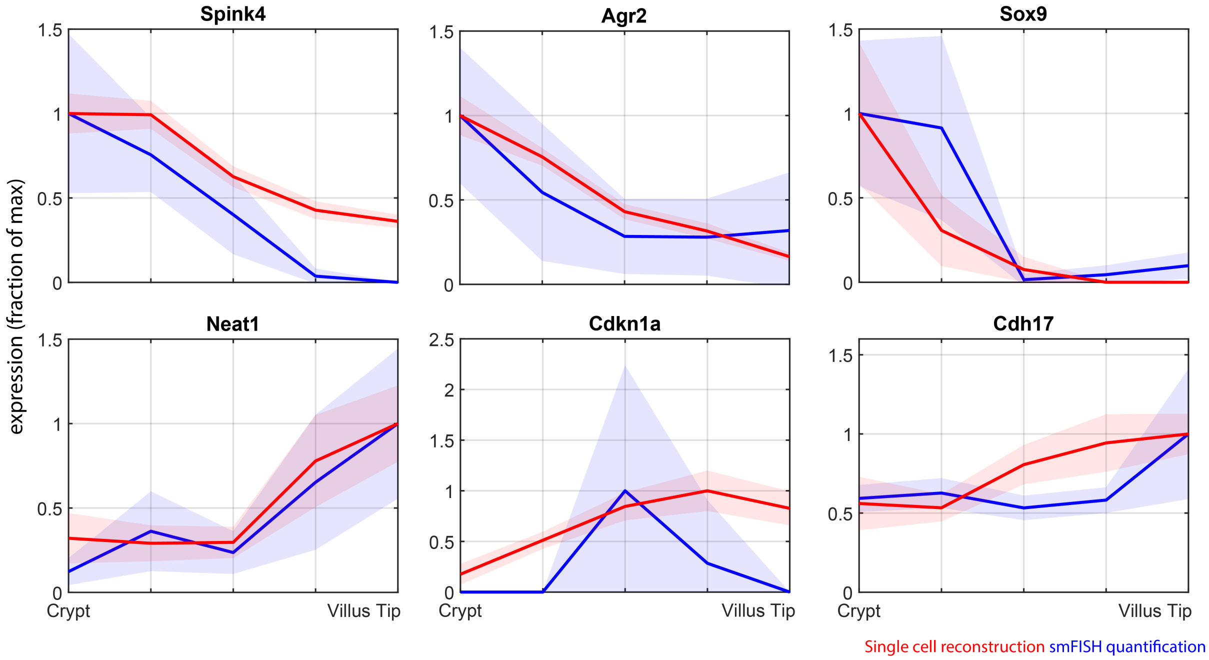

Supplementary Figure 8 Validation of the reconstructed zonation profiles using smFISH. Blue line - smFISH mean expression level, red line - reconstructed profile based on the single cell analysis. Light areas denote the SEM. SmFISH results based on 2 mice, 5 villi per mouse. Source data are provided as a Source Data file.

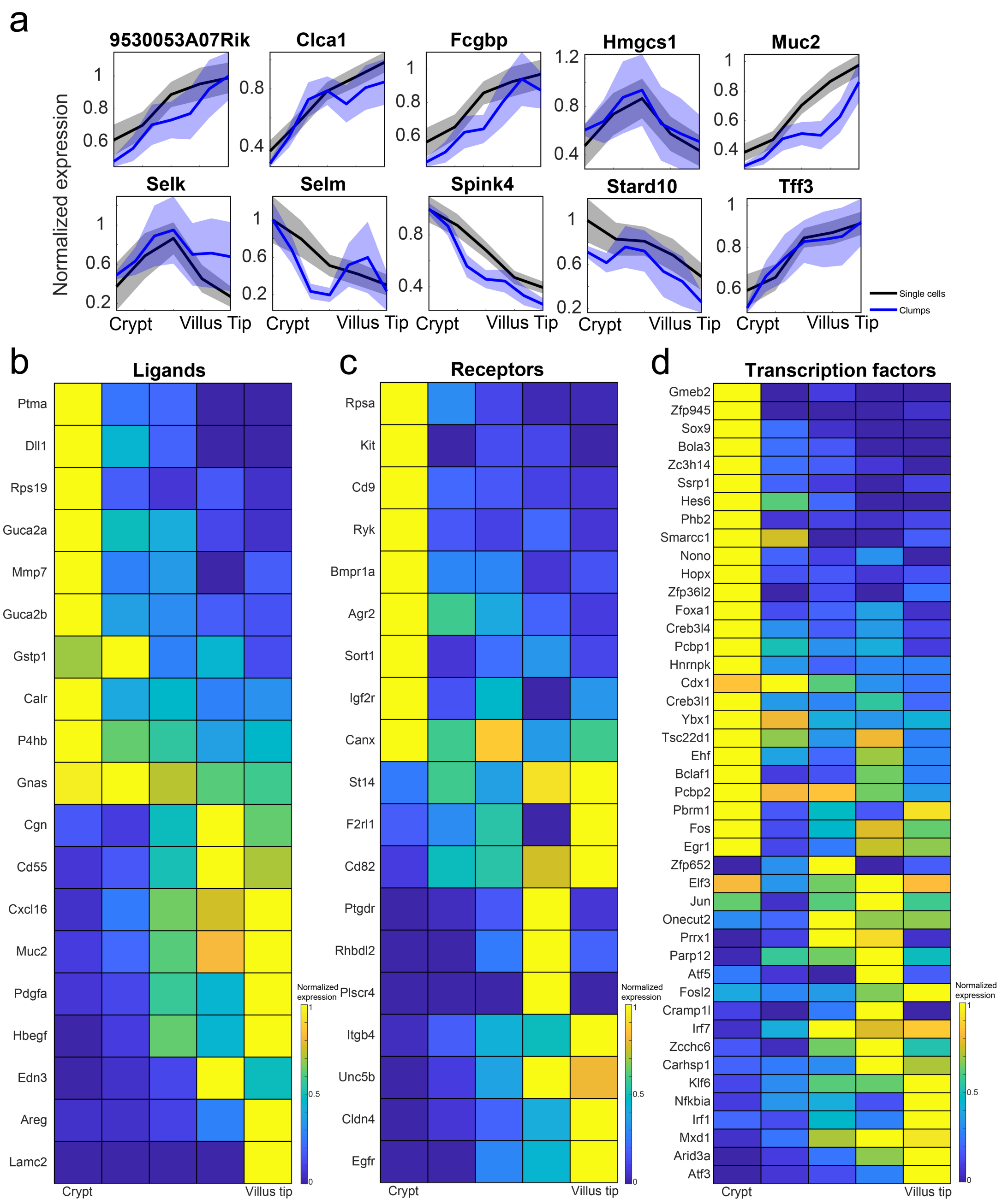

Supplementary Figure 9 (a) Validation of the reconstructed zonation profiles of goblet cells using clumps data. Blue line represents the reconstruction based on clumps mean expression level, black line - the reconstructed profile based on the single cell analysis. Light patches are the SEM. Profiles were smoothed with a moving average window of 3. (b-d) Zonated expression of (b) ligands, (c) receptors and (d) transcription factors in goblet cells. Only genes with expression above $5*10^{-5}$ and qval<0.25 are shown.

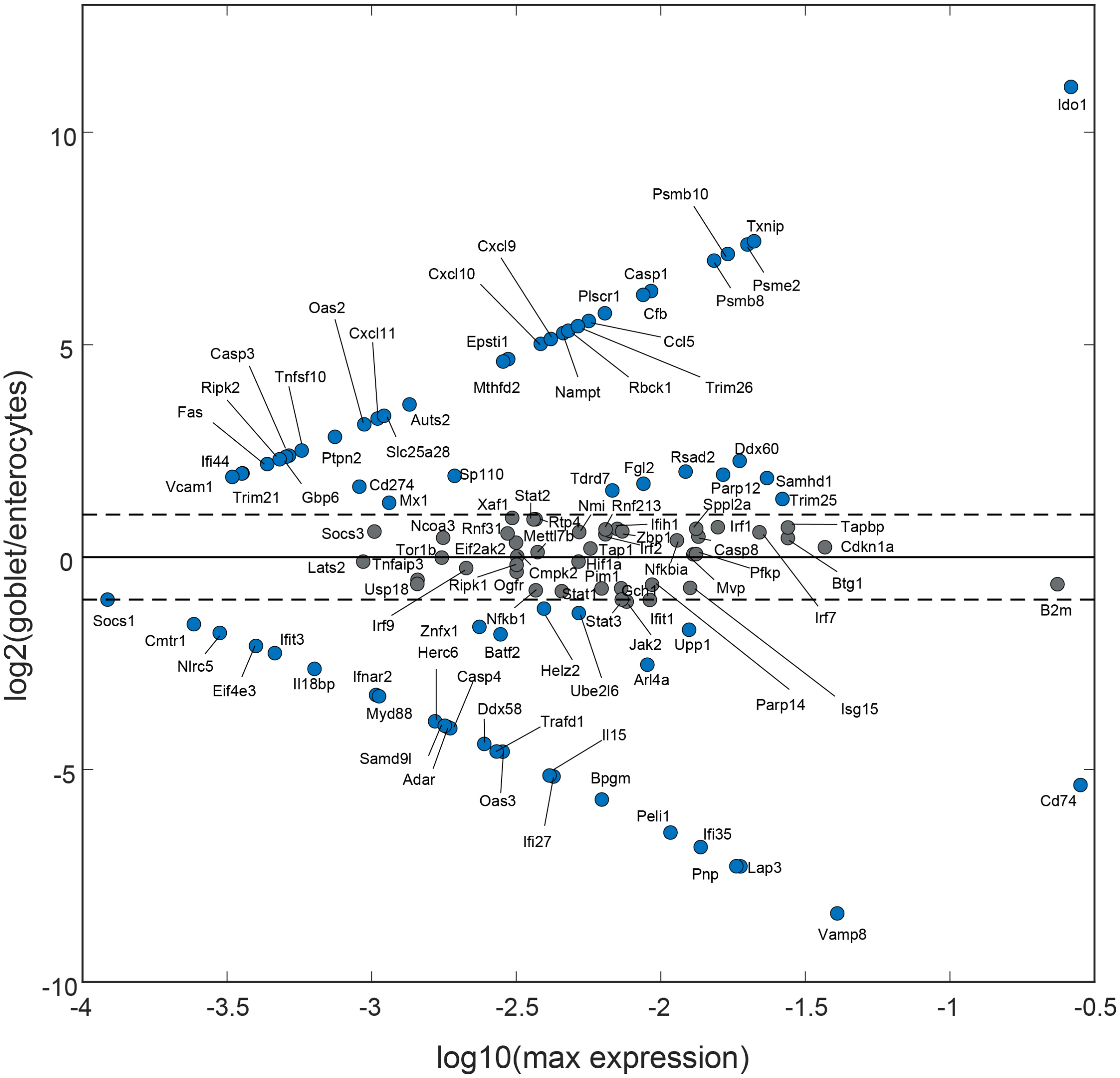

Supplementary Figure 10 Interferon-gamma response genes are differentially expressed between villus tip enterocytes, and villus tip goblet cells.

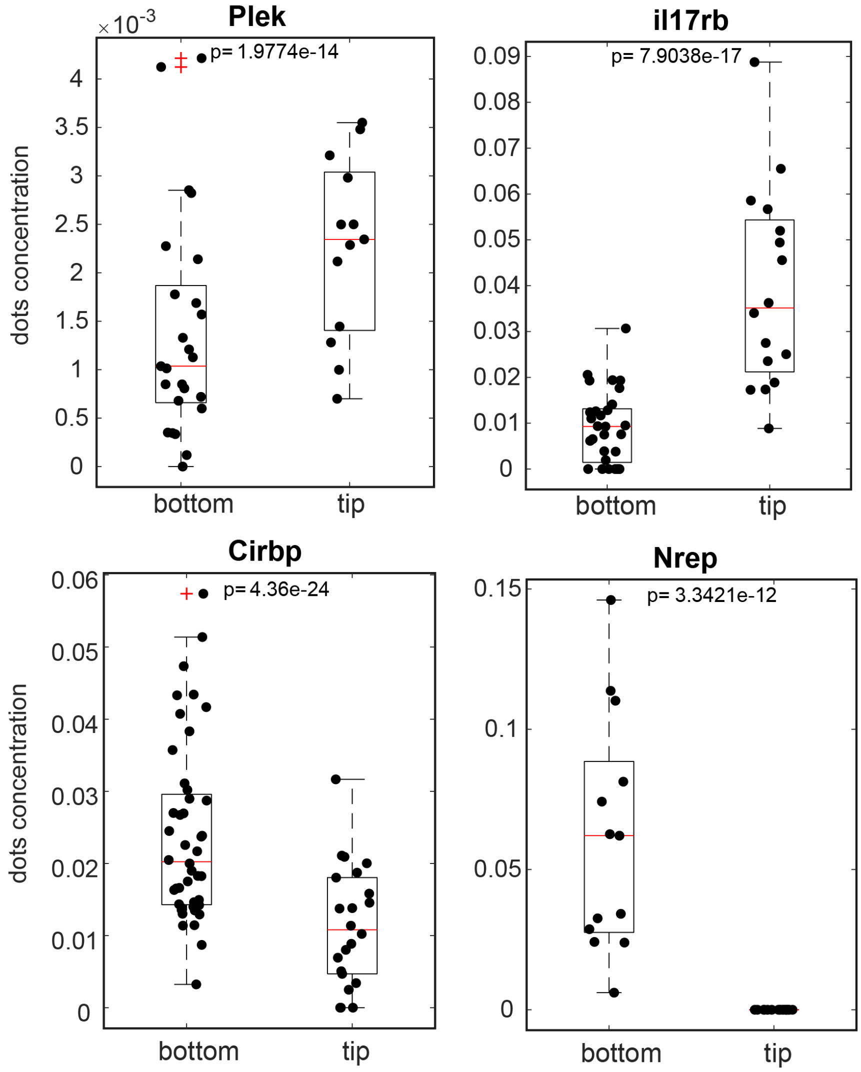

Supplementary Figure 11 smFISH quantification of zonated tuft genes. P value was calculated by Mann Whitney U test two-sided. n = 20 cells were examined over 2 mice. Red lines are medians, black boxes are 25-75 percentiles. Whiskers extend to the most extreme data point within 1.5× the interquartile range (IQR) from the box. Source data are provided as a Source Data file.

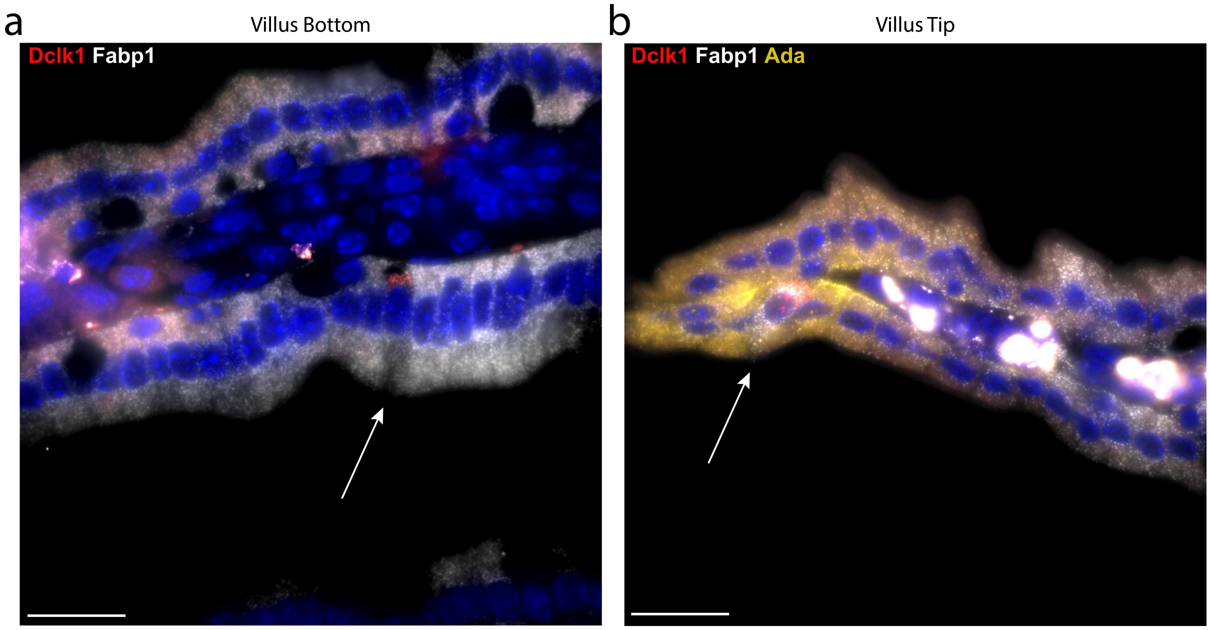

Supplementary Figure 12 whole images of the representative smFISH in figure 4 of the main manuscript (a) villus bottom (b) villus tip. Scale bar 30 μm. Arrows mark the tuft cells. (a-b) Image representative of n=20 cells over 2 mice.

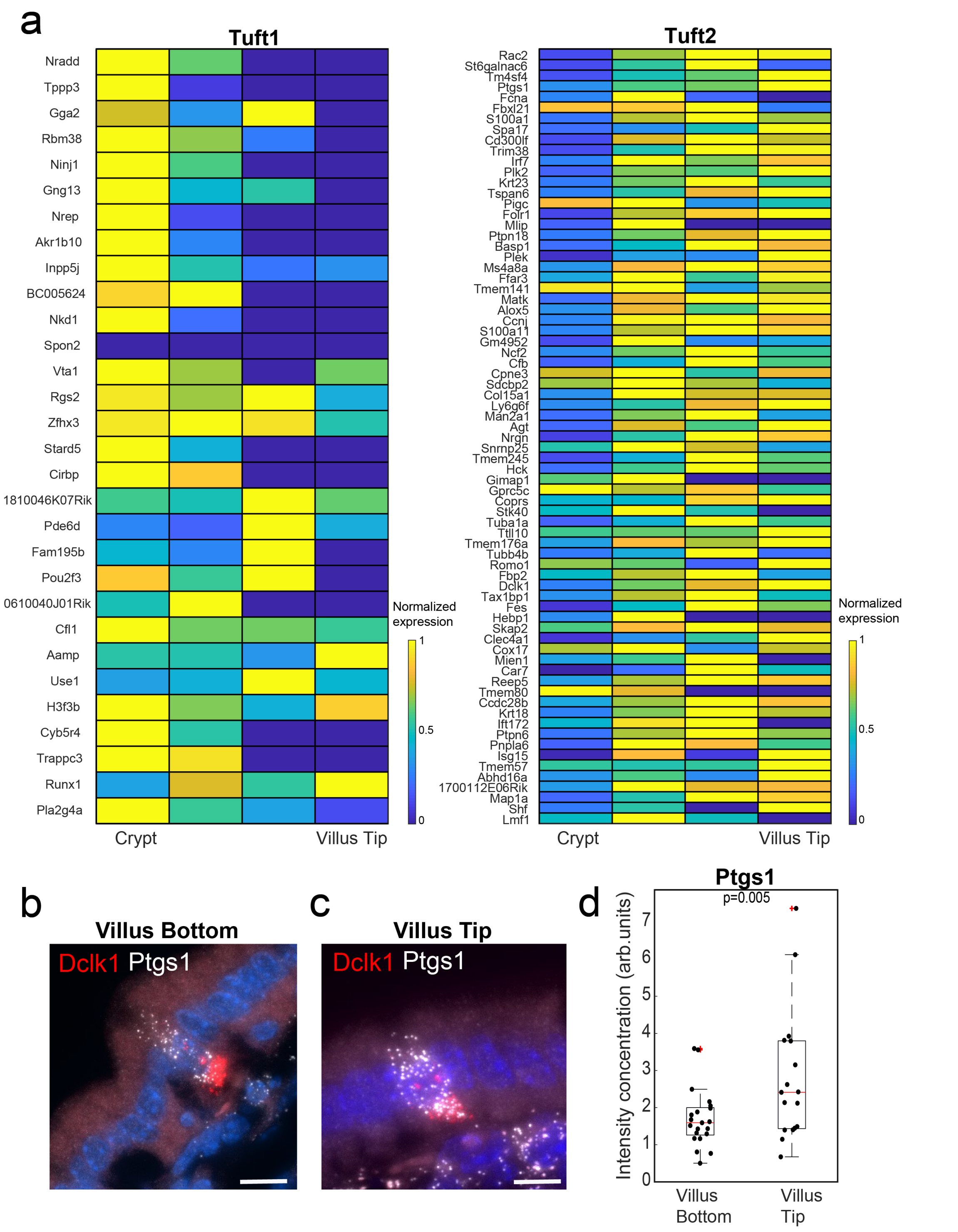

Supplementary Figure 13 (a) Heatmap of zonation profiles of genes specific for Tuft1 and Tuft2 classes. List of Tuft1 and Tuft2 genes was taken from the "consensus gene list" which appears in Supplementary Data 7 of Haber et. al.[17]. Profiles are normalized to their maximal value across the crypt-villus zones. (b-c) Representative smFISH images of the Tuft2 zonated gene Ptgs1 (white dots) in a villus bottom (left) and a villus tip (right) tuft cell. Tuft cells were identified by Dclk1 expression (red). Scale bar 15μm. (d) Quantification of Ptgs1 smFISH experiment. P value was calculated by Mann Whitney U test two-sided. n =

20 cells, 2 mice. Red lines are medians, black boxes are 25-75 percentiles. Whiskers extend to the most extreme data point within 1.5× the interquartile range (IQR) from the box. Source data are provided as a Source Data file.

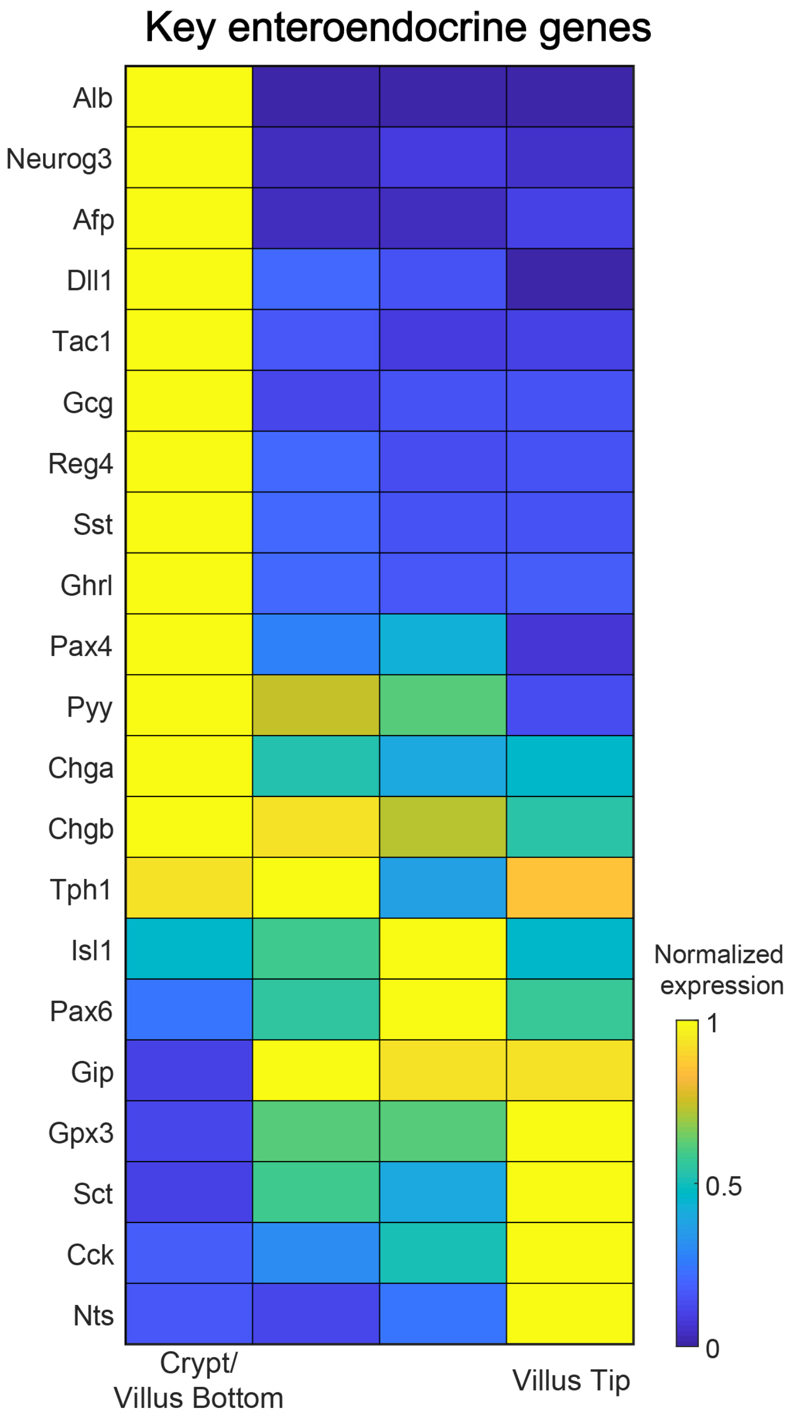

Supplementary Figure 14 Heatmap of zonation profiles of genes encoding key enteroendocrine genes. Profiles are normalized to their maximal value across the crypt-villus zones.

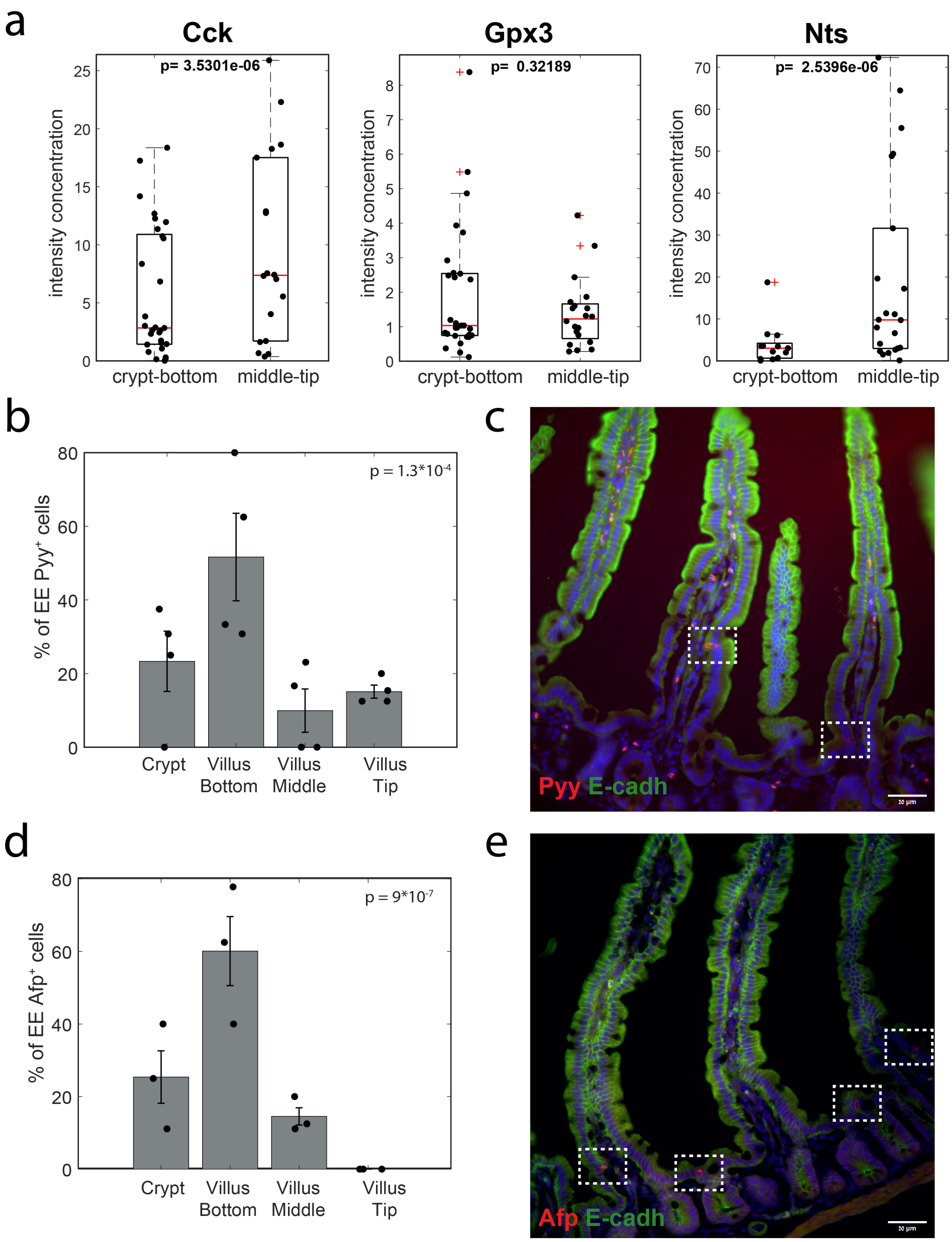

Supplementary Figure 15 (a) SmFISH quantification of zonated enteroendocrine genes. Red lines are medians, black boxes are 25-75 percentiles. Whiskers extend to the most extreme data point within 1.5× the interquartile range (IQR) from the box. P value was calculated by Mann Whitney U test two-side. n=3-4 mice. (b,d) Quantification of Pyy+ and Afp+ enteroendocrine cells in crypt and villus bottom, middle and tip over 3-4 mice. P values are Fisher exact tests (two-sided) for the frequencies of the cells between the two lower zones and two upper zones. Representative smFISH images of the enteroendocrine crypt-zonated gene (c) Pyy (red) and (e) Afp (red). (c,e) Image representative of n= 3-4 mice. Source data are provided as a Source Data file.

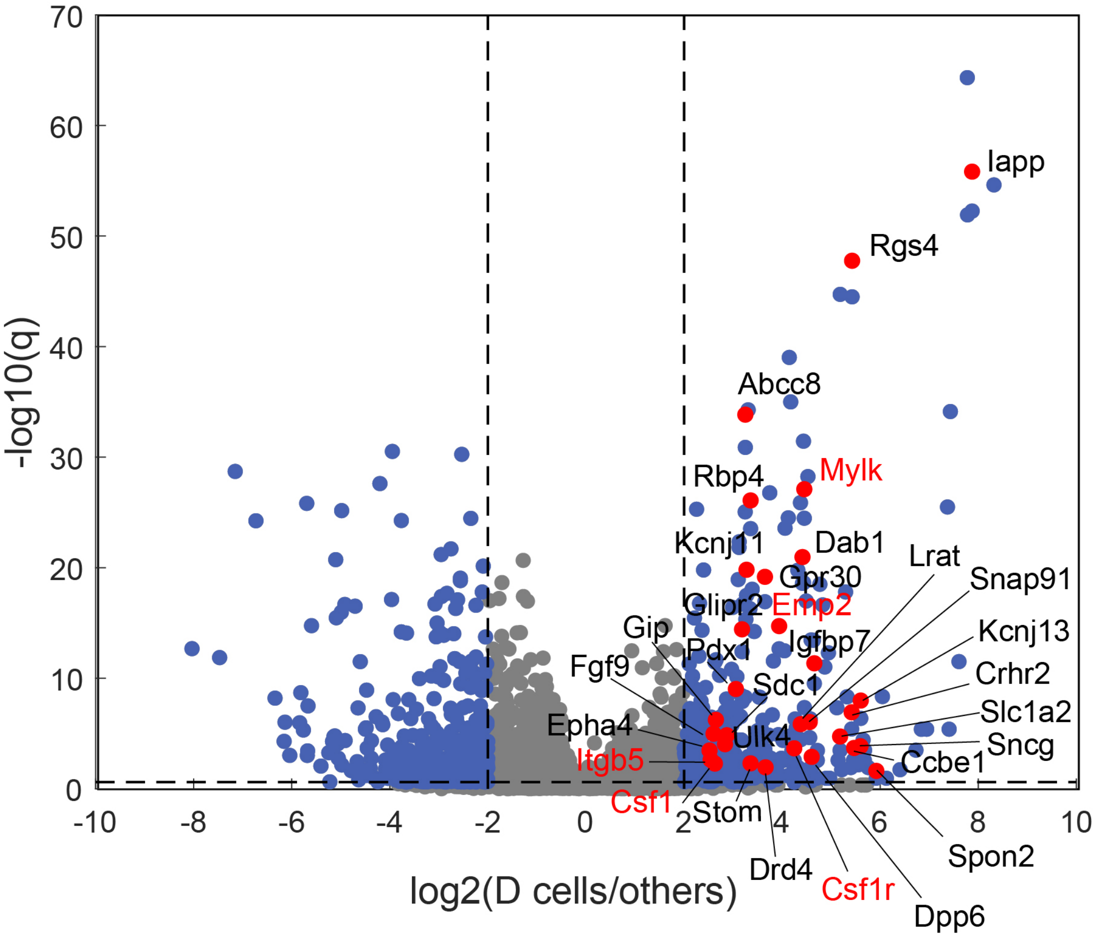

Supplementary Figure 16 Differential gene expression (DGE) analysis between D cells and EC and L cells. Volcano plot of DGE between D cells and EC and L cells. Labeled dots are selected differentially expressed genes related to GO adhesion program.

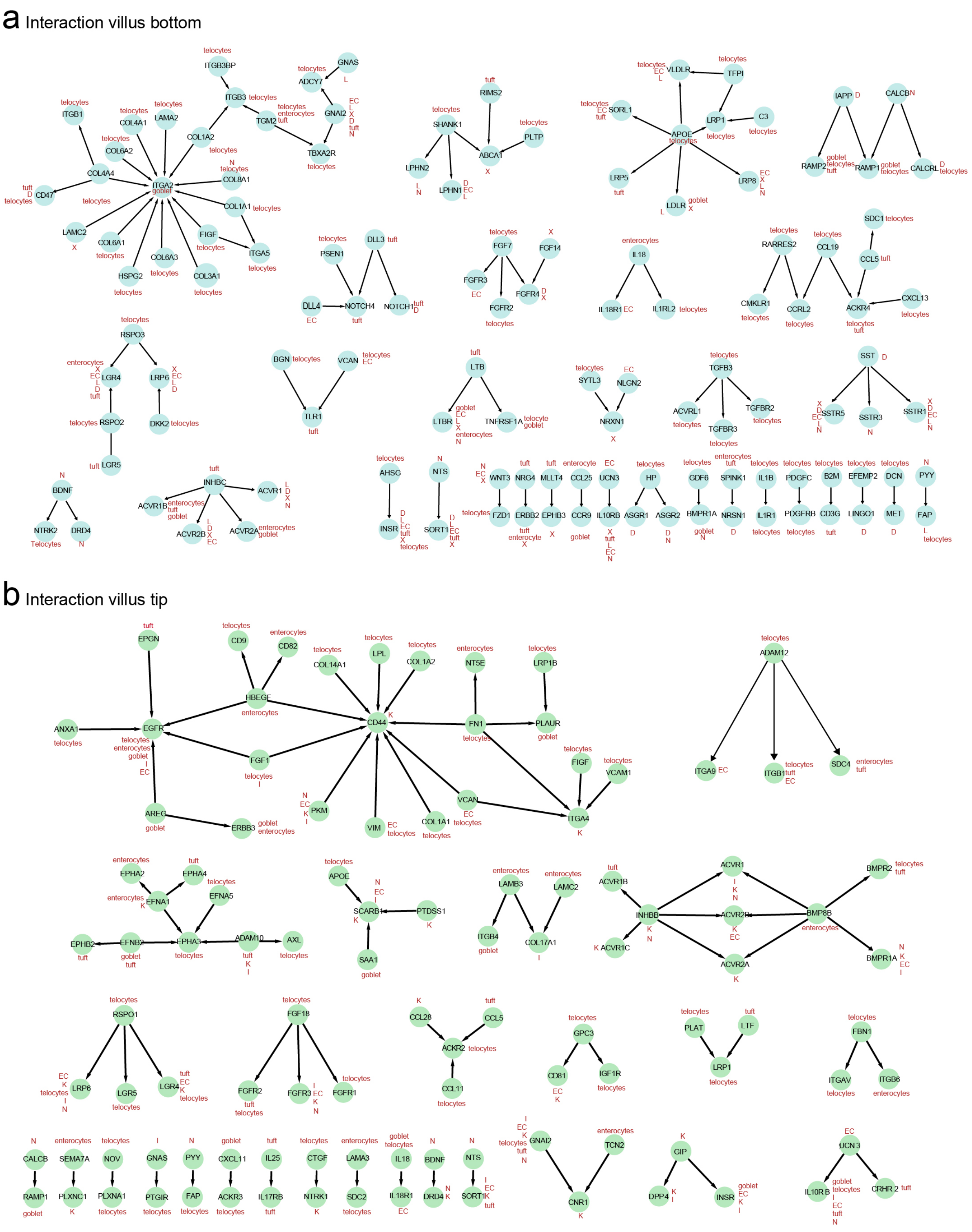

Supplementary Figure 17 Networks of enriched ligand-receptor interactions between epithelial and mesenchymal cells (a) at the villus bottom and (b) at the villus tip. Expression of either the ligand or the receptor above 2*10^{-5} and $\boldsymbol{Z_{interaction}}$ higher than 5 (Methods).

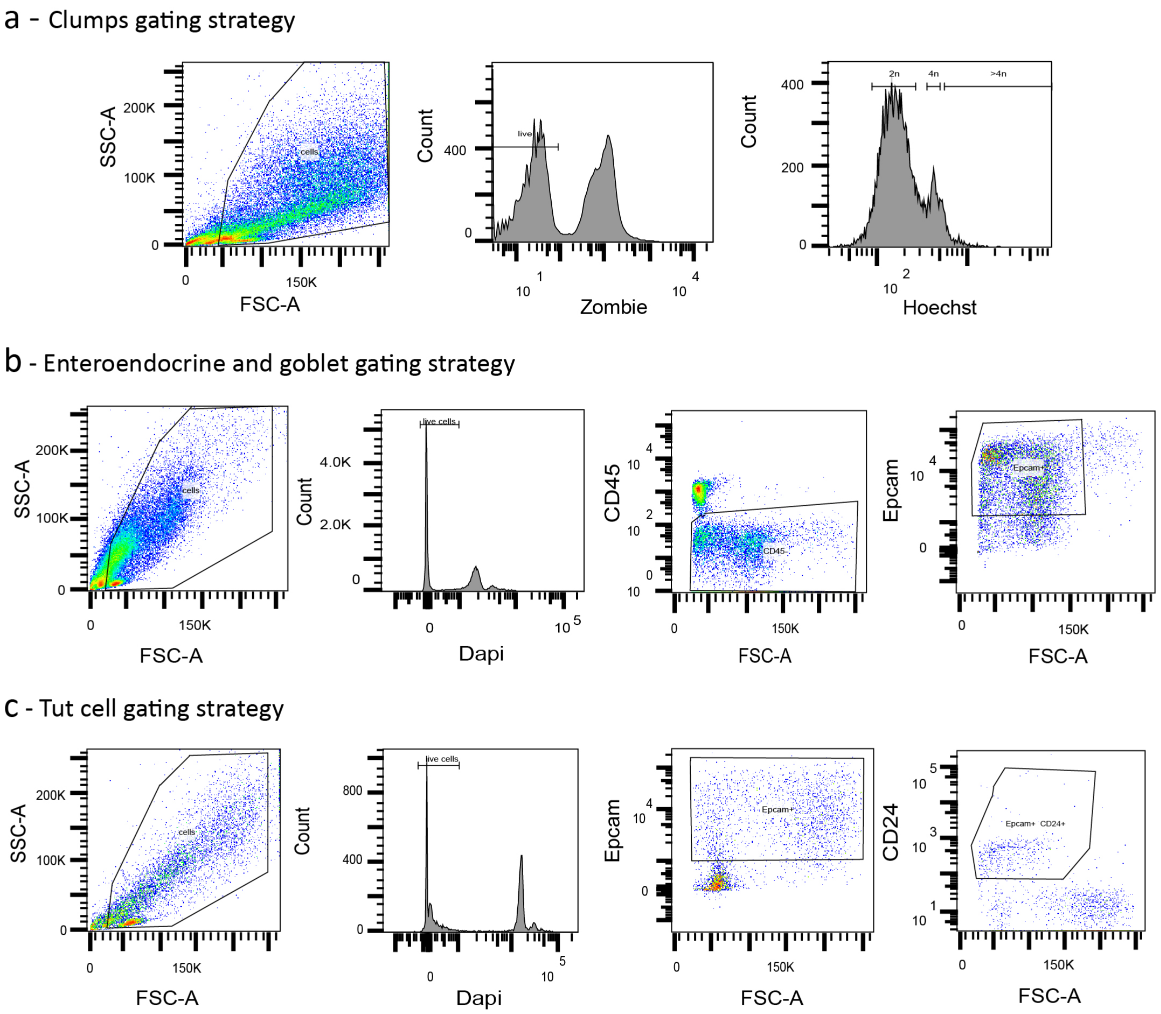

Supplementary Figure 18 FACS gates used to enrich for (a) clumps, (b) enteroendocrine and goblet single cells and (c) tuft cells. For clumps sorting, dead cells were excluded using the Zombie green staining and clumps were sorted based on Hoechst histogram. For single cell sorting, dead cells were excluded on the basis of Dapi incorporation. To enrich for enteroendocrine cells, cells were gated on CD45^{-} Epcam^{+}. Since tuft cells express CD45, to enrich for those, cells were gated only on Epcam^{+} CD24^{+}.
