# Supplementary material for: Clump sequencing exposes the spatial expression programs of intestinal secretory cells
Source: Nat Commun. 2021 May 24;12:3074. doi: 10.1038/s41467-021-23245-2 (PMC8144370; doi:10.1038/s41467-021-23245-2)
Supplement: Supplementary file 3 — Description of Additional Supplementary Files [file 41467_2021_23245_MOESM3_ESM.docx]

Description of Additional Supplementary Files

Title: Supplementary Data 1.

Description: Landmark genes table

Title: Supplementary Data 2.

Description: Clumps zonation table. P values were calculated with the

Kruskal -Wallis test, q values adjusted for multiple comparisons were calculated using

the Benjamini and Hochberg method (implemented in the MATLAB function mafdr),

applied to all genes for which maximal expression across zones exceeded 5*10-6.

Title: Supplementary Data 3.

Description: Single cell zonation table. P values were calculated with the

Kruskal -Wallis test, q values adjusted for multiple comparisons were calculated using

the Benjamini and Hochberg method (implemented in the MATLAB function mafdr),

applied to all genes for which maximal expression across zones exceeded 5*10-6.

Title: Supplementary Data 4.

Description: GSEA results

Title: Supplementary Data 5.

Description: Sequences of smFISH probes

Title: Supplementary Data 6.

Description: Mean expression by cell types

Title: Supplementary Data 7.

Description: Cell type markers

Title: Supplementary Data 8.

Description: Proportions of secretory cells

Title: Supplementary Data 9.

Description: GO results

Title: Supplementary Data 10.

Description: Ligand-receptor analysis

Title: Supplementary Data 11.

Description: Robustness analysis parameters
