# Supplementary material for: Clump sequencing exposes the spatial expression programs of intestinal secretory cells
Source: Nat Commun. 2021 May 24;12:3074. doi: 10.1038/s41467-021-23245-2 (PMC8144370; doi:10.1038/s41467-021-23245-2)
Supplement: Supplementary file 15 — Reporting Summary [file 41467_2021_23245_MOESM15_ESM.pdf]

nature research

Corresponding author(s): Shalev Itzkovitz

Last updated by author(s): Apr 7, 2021

Reporting Summary

Nature Research wishes to improve the reproducibility of the work that we publish. This form provides structure for consistency and transparency in reporting. For further information on Nature Research policies, see our Editorial Policies and the Editorial Policy Checklist.

Statistics

For all statistical analyses, confirm that the following items are present in the figure legend, table legend, main text, or Methods section.

n/a	Confirmed	
☐	☒	The exact sample size (*n*) for each experimental group/condition, given as a discrete number and unit of measurement
☐	☒	A statement on whether measurements were taken from distinct samples or whether the same sample was measured repeatedly
☐	☒	The statistical test(s) used AND whether they are one- or two-sided<br>*Only common tests should be described solely by name; describe more complex techniques in the Methods section.*
☐	☒	A description of all covariates tested
☐	☒	A description of any assumptions or corrections, such as tests of normality and adjustment for multiple comparisons
☐	☒	A full description of the statistical parameters including central tendency (e.g. means) or other basic estimates (e.g. regression coefficient) AND variation (e.g. standard deviation) or associated estimates of uncertainty (e.g. confidence intervals)
☐	☒	For null hypothesis testing, the test statistic (e.g. *F*, *t*, *r*) with confidence intervals, effect sizes, degrees of freedom and *P* value noted<br>*Give P values as exact values whenever suitable.*
☒	☐	For Bayesian analysis, information on the choice of priors and Markov chain Monte Carlo settings
☒	☐	For hierarchical and complex designs, identification of the appropriate level for tests and full reporting of outcomes
☐	☒	Estimates of effect sizes (e.g. Cohen's *d*, Pearson's *r*), indicating how they were calculated

Our web collection on statistics for biologists contains articles on many of the points above.

Software and code

Policy information about availability of computer code

Data collection	We used BD FACSDiva'" Software 8.0.1 (BD Biosciences-US) for sorting.<br>smFISH imaging was done on the NIS element software AR 5.11.01
Data analysis	For RNA seq analysis, we used BCL2fasq tool v2.20.0.422(Illumina), STAR (2.5.3a) for alignment and zUMIs for quantification. The softwares used to analyze the data are Matlab 2019a, R 3.6.1 and RStudio 1.2.5019, ImageM 0.31, IDEAS 6.2, Seurat v3.2package,, FIJI win64.<br>Probes libraries were designed using Stellaris FISH Probe Designer Software (Biosearch Technologies, Inc., Petaluma, CA)<br>For manuscripts utilizing custom algorithms or software that are central to the research but not yet described in published literature, software must be made available to editors

For manuscripts utilizing custom algorithms or software that are central to the research but not yet described in published literature, software must be made available to editors and reviewers. We strongly encourage code deposition in a community repository (e.g. GitHub). See the Nature Research guidelines for submitting code & software for further information.

Data

Policy information about availability of data

All manuscripts must include a data availability statement. This statement should provide the following information, where applicable:
- Accession codes, unique identifiers, or web links for publicly available datasets
- A list of figures that have associated raw data
- A description of any restrictions on data availability

Data generated in this study has been deposited in Gene Expression Omnibus with the accession code: GSE154714. Single cell dataset also includes epithelial cells from GSE13447943. Enteroendocrine single cell dataset was acquired from NCBI GEO dataset browser, with accessions code: GSE11356112.

Field-specific reporting

Please select the one below that is the best fit for your research. If you are not sure, read the appropriate sections before making your selection.

[x] Life sciences   [ ] Behavioural & social sciences   [ ] Ecological, evolutionary & environmental sciences

For a reference copy of the document with all sections, see nature.com/documents/nr-reporting-summary-flat.pdf

Life sciences study design

All studies must disclose on these points even when the disclosure is negative.

Sample size	The sample sizes (number of mice and total number of sequenced cells) where chosen as to be sufficient to obtain sufficient power to discern gene expression differences between the crypt-villus axis.
Data exclusions	For clumps, we excluded clumps containing less than 200 genes and with over 30% mitochondrial genes.<br>For single cells, cells with either total UMI counts lower than 200 or higher than 7,000 or total gene counts lower than 150 or higher than 1500 or mitochondrial content of over 40% were removed.
Replication	We have used 2-5 biological replicate for each experiment - all attempts at replication were successful as there was no sample which was excluded due to outliers.
Randomization	No allocation for experimental groups in our study. All mice underwent same procedure
Blinding	N/A - all mice were treated the same

Reporting for specific materials, systems and methods

We require information from authors about some types of materials, experimental systems and methods used in many studies. Here, indicate whether each material, system or method listed is relevant to your study. If you are not sure if a list item applies to your research, read the appropriate section before selecting a response.

Materials & experimental systems

n/a	Involved in the study	
[ ]	[x]	Antibodies
[x]	[ ]	Eukaryotic cell lines
[x]	[ ]	Palaeontology and archaeology
[ ]	[x]	Animals and other organisms
[x]	[ ]	Human research participants
[x]	[ ]	Clinical data
[x]	[ ]	Dual use research of concern

Methods

n/a	Involved in the study	
[x]	[ ]	ChIP-seq
[ ]	[x]	Flow cytometry
[x]	[ ]	MRI-based neuroimaging

Antibodies

Antibodies used	The following antibodies were used for sorting: Hoechst 33342 (15 ug ml-1), APC-anti-Epcam (1:100, BioLegend, 118214), PE/Cy7-anti-CD45 (1:1000, Biolengend, 103114), PE/Cy7-anti-CD24 (1:100, BioLegend, 101821
Validation	all antibodies were validated on manufacturer website.<br>APC-anti-Epcam (BioLegend, 118214), validated in previous refs, reported in manufacturer's page : https://www.biolegend.com/<br>enus/<br>products/apc-anti-mouse-cd326-ep-cam-antibody-4974;<br>PE/Cy7-anti-CD45 (Biolengend, 103114), for validation from website (https://www.biolegend.com/en-us/products/pe-cyanine7-<br>antimouse-<br>cd45-antibody-1903): "in this experiment we are looking at populations of immune cells in the bone marrow of mice. This antibody is used to stain CD45 for flowcytometry". "This antibody is great for staining CD45".<br>PE/Cy7-anti-CD24(BioLegend, 101821), validated in previous refs, reported in manufacturer's page https://www.biolegend.com/<br>engb/<br>search-results/pe-cyanine7-anti-mouse-cd24-antibody-3862

Animals and other organisms

Policy information about studies involving animals; ARRIVE guidelines recommended for reporting animal research

Laboratory animals	C57BL/6 8-12weeks old male mice, obtained from Envigo laboratories (Israel)
Wild animals	No wild animals were involved in this study
Field-collected samples	No field-collected samples were used in this study
Ethics oversight	Mouse experiments were approved by the Institutional Animal Care and Use Committee of the Weizmann Institute of Science and performed in accordance with institutional guidelines.

Note that full information on the approval of the study protocol must also be provided in the manuscript.

Flow Cytometry

Plots

Confirm that:

- [x] The axis labels state the marker and fluorochrome used (e.g. CD4-FITC).
- [x] The axis scales are clearly visible. Include numbers along axes only for bottom left plot of group (a 'group' is an analysis of identical markers).
- [x] All plots are contour plots with outliers or pseudocolor plots.
- [x] A numerical value for number of cells or percentage (with statistics) is provided.

Methodology

Sample preparation	Clumps dissociation - The Jejunum was harvested, flushed with cold 1X DPBS, laterally cut and incubated for 20 min on ice in a 10mM EDTA solution. Afterwards, the tissue was cut into 1cm pieces , moved in a pre-warmed 10 mM EDTA solution for 5 min and shaked vigorously at the end of the incubation time. Dissociated cells were collected and filtered through a 100 µm cell strainer. Cells were spun down at 300g for 5 min at 4 °C. Pellet was resuspended and incubated for nuclear staining for 5 min at RT in a solution of DMEM + 10% FBS + 10mM HEPES + Hoechst 33342 (15 µg ml-1). To prevent the cells from pumping out the Hoechst dye, Reserpine (5µM) was also added. Cells were resuspended in PBS and Alexa Fluo 488 Zombie Green (BioLegend) was added at a dilution of 1:500, to later enable the detection of viable cells by FACS. Cells were kept in a rotator in the dark at room temperature for 15 min. After spinning down (500g. for 5 min at 4 °C), cells were resuspended in FACS buffer (2 mM EDTA, pH 8, and 0.5% BSA in 1× PBS) at a concentration of 106 cells in 100 µl.<br><br>Single cell isolation – To obtain single cell suspension, rather than clumps, the tissue was incubated for 10 min on ice in a 10mM EDTA solution, before to be cut in small pieces and moved for other 10 min in a pre-warmed 10 mM EDTA solution. The tissue was shaked vigorously every 2 min. Cells were filtered through a 70 µm cell strainer and spun down at 300g for 5 min at 4 °C. Cells were resuspended in FACS buffer and stained with combination of APC-anti-Epcam (BioLegend, 118214) and PE/Cy7-anti-CD45 (Biolengend, 103114) or APC-anti-Epcam and PE/Cy7-anti-CD24 (BioLegend, 101821). FcX blocking solution (BioLegend) was added at a dilution of 1:50.
Instrument	SORP-FACSAriaII sorter (BD)
Software	BD FACSDiva™ Software 8.0.1 | BD Biosciences-US, Flow-Jo
Cell population abundance	Abundance in clumps sorting: doublets 2%, clumps 7.8%
Gating strategy	Cells were sorted using SSC and FSC gates. For clumps sorting, dead cells were excluded using the Zombie green staining and clumps were sorted based on Hoechst histogram. For single cell sorting, dead cells were excluded on the basis of Dapi incorporation. Sorted cells were negative for CD45 and positive for Epcam. To enrich for enteroendocrine cells, cells were gated on CD45- Epcam+. Since tuft cells express CD4517, to enrich for those, cells were gated only on Epcam+ CD24+.

- [x] Tick this box to confirm that a figure exemplifying the gating strategy is provided in the Supplementary Information.
